# Peptides for Health Benefits 2019

**DOI:** 10.3390/ijms21072543

**Published:** 2020-04-06

**Authors:** Cristina Martínez-Villaluenga, Blanca Hernández-Ledesma

**Affiliations:** 1 Institute of Food Science, Technology and Nutrition (ICTAN-CSIC), Juan de la Cierva 3, 28006 Madrid, Spain; c.m.villaluenga@csic.es; 2 Institute of Food Science Research (CIAL, CSIC-UAM, CEI UAM+CSIC), Nicolás Cabrera, 9, 28049 Madrid, Spain

In recent years, peptides have received increased interest in pharmaceutical, food, cosmetics and various other fields. The high potency, specificity and good safety profile are the main strengths of bioactive peptides as new and promising therapies that may fill the gap between small molecules and protein drugs. Peptides possess favorable tissue penetration and the capability to engage into specific and high-affinity interactions with endogenous receptors. These positive attributes of peptides have driven research in evaluating peptides as versatile tools for drug discovery and delivery. In addition, among bioactive peptides, those released from food protein sources have acquired importance as active components in functional foods and nutraceuticals because they are known to possess regulatory functions that can lead to health benefits.

This Special Issue of *International Journal of Molecular Sciences* represents the second in a series dedicated to peptides. This issue includes thirty-six outstanding papers describing examples of the most recent advances in peptide research and its applicability. 

The Special Issue begins with a group of papers exploring aspects of synthetic peptides that are of significance to develop novel drugs for controlling and/or managing chronic diseases. It begins with a study of Gaglione et al. [1] on the identification of three cryptides in human apolipoprotein B and evaluation of their antimicrobial and anti-biofilm properties individually or in combination with ciprofloxacin towards *Pseudomonas* and *Burkholderia* strains clinically isolated from cystic fibrosis patients. These findings will open interesting perspectives to apoB cryptides applicability in the treatment of chronic lung infections associated with cystic fibrosis disease. The issue follows with research by Tarallo et al. [2] on a new tetrameric tripeptide inhibitor of vascular endothelial growth factor receptor 1 that exerts anti-angiogenic activity at ocular level by oral delivery in a preclinical model of age-related macular degeneration. Asai et al. [3] demonstrate that Pro-Hyp and Hyp-Gly play crucial roles in proliferation of fibroblasts attached on collagen gel. Russjan and Kaczynska [4] investigate the beneficial effects of neurotensin in murine model of hapten-induced asthma. In another paper, Russjan et al. [5] investigate the anti-inflammatory potency of hybrid peptide-PK20, composed of neurotensin and endomorphin-2 pharmacophores in a mouse model of non-allergic asthma. Improved anti-inflammatory potency of the hybrid over the mixture of its moieties shows potential as a promising tool in modulating airway inflammation in asthma. Pershina et al. [6] study the gender specific effects of a pegylated glucagon-like peptide 1 (GLP-1), used in the treatment regime for metabolic disorder and chronic obstructive pulmonary disease. Oludiran et al. [7] demonstrate that potency of antimicrobial piscidin peptides depends on environmental oxygen, therefore, the development of pharmaceuticals from host-defense peptides such as piscidin will necessitate consideration of oxygen levels in the targeted tissue. The chemokine-like activity of the synthetic dipeptide pidotimod is studied by Caccuri et al. [8]. The study also defines the mechanism of action for chemokine-like activity of pidotimod and points on the possible role that this synthetic dipeptide may play in leukocyte trafficking and function. The potency, toxicity and mechanisms of action of Ps-K18 is examined by Jang et al. [9] aiming to develop antibiotics derived from bioactive peptides for the treatment of Gram-negative sepsis. Golda et al. [10] screen a library of synthetic peptides to identify those with antibacterial potential against multidrug-resistant *Staphylococcus aureus.* The bactericidal and keratinocytes cytoprotective mechanisms against invading bacteria are also elucidated. *Staphylococcus aureus* and *Pseudomonas aeruginosa*, individually or in co-occurrence, are the two main pathogens implied in multiple bacterial infections. Since their discovery, the antimicrobial peptides (AMPs) of innate defense have been considered as a potential alternative to conventional antibiotics. However, no commercial AMPs are still available. The review of Rončević et al. [11] is aimed at describing these peptides, their mechanisms of action, their biological and biophysical properties as well as the developed models to design and produce new molecules with high antimicrobial potency and low toxicity. Intragenic antimicrobial peptide Hs02 is demonstrated by Bessa et al. [12] to exert antimicrobial properties against *Pseudomonas aeruginosa* and *Staphylococcus aureus*, also hampering the proliferation of their single and dual-species biofilms. The study of Prasad et al. [13] reviews the role of host defense peptides in different inflammatory conditions and diseases, associating this role with the physicochemical properties of peptides and their interaction with various receptors that define their immunomodulatory effects. In another paper, Nácher-Juan et al. [14] investigate the role of peptide osteostatin derived from parathyroid hormone-related protein against rheumatoid arthritis. This peptide, administered to collagen-induced arthritic mice, decreases the severity of the disease through modulation of immune and inflammatory biomarkers. Palus et al. [15] report the neurothropic and/or neuroprotective properties of galanin, alone or in combination with other neuroactive substances such as vasoactive intestinal peptide, neuronal nitric oxide synthase and cocaine- and amphetamine-regulated transcript peptide in the recovery processes in the stomach enteric nervous system neurons following acrylamide intoxication. The extra domain B of fibronectin (EDB-FN) localized in the extracellular matrix can differentiate aggressive prostate cancer from benign prostatic hyperplasia. Park et al. [16] synthesize two cyclic peptides, CTVRTSADC and KTVRTSADE with ability to target EDB-FN, and develop different conjugates with anticancer drugs docetaxel and doxorubicin. The conjugates show selective cytotoxic effects against prostate cancer cells without affecting normal prostate cells.

Following, there is a short series of articles dealing with the elucidation of modes of action of known food-derived bioactive peptides. Fernández-Tomé et al. [17] provide new evidence on the chemopreventive activity of peptide lunasin, a bioactive peptide from soybean and other vegetal sources, on colorectal cancer by modulating both the parental and the tumorsphere-derived subsets of HCT-116 cells. The underlying molecular mechanisms behind the inhibitory effects of lunasin on cell cycle progress of colon cancer cells and cytotoxicity were also discussed. Martínez-Sánchez et al. [18] describe the beneficial effects of dry-cured ham peptides previously identified to prevent from endothelial dysfunction and inflammation. In silico dockings show the predicted modes of binding of four bioactive peptides with the regulatory subunit NEMO of the NF-κB transcription factor and angiotensin I converting-enzyme.

Another group of papers explores the potential of new proteins as sources of bioactive peptides. Cai et al. [19] explore the cytoprotective mechanism of antioxidant pentapeptides from a protein hydrolysate of miiuy croaker (*Miichthys miiuy*) swim bladder against oxidative damage to human umbilical vein endothelial cells. Gomez et al. [20] report on the potential bioactivities of Portuguese oyster (*Crassostrea angulata*) proteins through in silico analyses and in vitro tests. *C. angulata* proteins were proven to be sources of angiotensin I-converting enzyme and dipeptidyl peptidase IV inhibitory peptides with pharmaceutical and nutraceutical applications. Using different commercial proteases, Ding et al. [21] produce hydrolysates from velvet antler with antioxidant properties. The protective effect against oxidative stress of a tetrapeptide produced by Alcalase is investigated in Chang liver cells and a zebrafish model. León-Lopez et al. [22] describe the biochemical, structure and physico-chemical features as well as the antioxidant activity of collagen hydrolysates from sheepskins. A soybean product obtained after combined hydrolysis with Prozyme and fermentation with *Lactobacillus rhamnosus* EBD1 by Daliri et al. [23] show antihypertensive properties in both in vitro and in vivo models, without losing its activity after simulating its digestion by gastrointestinal enzymes. Peptides PPNNNPASPSFSSSS, GPKALPII and IIRCTGC, in which angiotensin-converting enzyme inhibitory activity had been previously demonstrated, are included in the soy product. Another review of Brady et al. [24] summarizes the antibacterial and anti-inflammatory activities of cecropins, a group of naturally occurring antimicrobial peptides found in insects. The strategies designed to overcome the existing limitations linked to their costly large-scale production and their use as therapeutic agents are also described.

The issue includes some studies on bioinformatic and proteomic tools useful for peptide research. Using molecular docking, Chamata et al. [25] describe the structure-activity relationships of peptide sequences present in whey/milk protein hydrolysates with high angiotensin converting enzyme inhibitory activity to a better understanding and prediction of their in vivo antihypertensive activity. Minkiewicz et al. [26] review the new opportunities offered by the BIOPEP-UWM database of bioactive peptides that include the possibility of annotating peptides containing D-enantiomers of amino acids, batch processing option, converting amino acid sequences into SMILES code, new quantitative parameters characterizing the presence of bioactive fragments in protein sequences and finding proteinases that release particular peptides. Using yeast proteome microarrays, Shah et al. [27] identify a total of 140 and 137 intracellular protein targets of antifungal peptides of Lactoferricin B and Histatin-5, respectively. The usefulness of this proteomic tool to find synergistic actions of bioactive peptides is also addressed. The in silico analysis carried out by Tejano et al. [28] reveal the role of *Chlorella sorokiniana* proteins as source of bioactive peptides. The BIOPEP’s profile shows that these proteins have multiple dipeptydil peptidase IV inhibitors, glucose uptake stimulants, antioxidant, regulating, anti-amnestic and anti-thrombotic peptides. Pepsin, bromelain and papain are the main proteases responsible for the release of bioactive peptides with pharmaceutical and nutraceutical potential. The review of Bozovičar and Bratkovic [29] focuses on recombinant peptide libraries useful for pharmaceutical industry in the drug discovery and delivery. These authors discuss different platforms for the display and/or expression of bioactive peptides as well as various diversification strategies for library design.

Another group of papers explores the effects of endogenous peptides on body functions and their potential for new drug alternatives. In a glioma mouse model, Kucheryavykh et al. [30] reveal by ELISA and immunofluorescence images that innate amyloid beta (Aβ) peptide is accumulated in glioma tumors and nearby blood vessels. Interestingly, the amyloidogenic Aβ peptide is co-localized with the lipid-free apolipoprotein E (apoE) in amyloid plaques in Alzheimer’s disease, where the apoE4 isoform plays a crucial role for the late onset disorder. In the study of Tsiolaki et al. [31], apoE peptide-analogues serve to predict the dynamics of apoE and apoE-Aβ complexes. The homeostasis of the organism is maintained by coordinated neuroendocrine and immune systems. Vasoactive intestinal peptide (VIP) is an endogenous neuropeptide produced by both neurons and endocrine and immune cells. Martínez et al. [32] review the biology of VIP and VIP receptor’s signaling and their protective immunomodulatory effects. The current evidence on strategies improving the stability, selectivity and effectiveness of VIP receptors analogs, the advances on new routes of administration and the potential clinical benefits against inflammatory and autoimmune disorders is described. Another neuropeptide described in the Special Issue is the prolactin-releasing peptide (PrRP). The anorexigenic neuroprotective effects of this peptide are reviewed by Pražienková et al. [33]. These authors also describe its therapeutic potential mediated by its actions on cardiovascular system, pain and stress. G-protein-coupled-seven-transmembrane receptors (GPCRs) are known by their modulatory properties of myeloid cell trafficking in microbial infections, inflammation, immune response and cancer progression. The review of Krepel and Wang [34] shows the existing evidence on one of these receptors from murine origin, called Fpr2, and its endogenous agonist peptide, cathelicidin-related antimicrobial peptide. Both are implied in normal mouse colon epithelial growth, repair and protective actions against inflammation-associated tumorigenesis.

Finally, a couple of articles describe new developed techniques to investigate the response of immune system. Thus, Kametani et al. [35] describe humanized mouse systems possessing immune cells as successful models to in vivo investigate the human immunity and predict the antibody response and immune adverse effects. Similarly, immune responses can be studied using an in situ mayor histocompatibility complex tetramer staining. As described by Abdelaal et al. [36], this technique, combined with immunohistochemistry, is a valuable tool for studying the Ag-specific T cell immune response in tissues. Combined techniques enable determining the localization, abundance and phenotype of T cells and characterizing Ag-specific T cells in specific tissues. Current applications in microbial infections, cancer and autoimmunity are also reviewed.

We wish to thank the invited authors for their interesting and insightful contributions, and look forward to a new set of advances in the bioactive peptides field to be included in the following Special Issue “Peptides for Health Benefits 2020” (https://www.mdpi.com/journal/ijms/special_issues/peptides_2020).
